# Editorial: Growth Hormone in Fertility and Infertility: Physiology, Pathology, Diagnosis and Treatment

**DOI:** 10.3389/fendo.2021.621722

**Published:** 2021-01-28

**Authors:** Jan Tesarik, John L. Yovich, Yves Menezo

**Affiliations:** ^1^ MARGen Clinic, Granada, Spain; ^2^ PIVET Medical Center, Perth, WA, Australia; ^3^ Laboratoire Clement, Paris, France

**Keywords:** growth hormone, fertility, infertility, physiology, pathology, diagnosis, treatment

## Introduction

Growth hormone (GH) has been used in the treatment of infertility since the late 1980s (reviewed in Homburg and Ostergaard (1)). However, its indication was mostly empirical, rather than scientifically founded, but with clearest benefits in women with hypopituitarism One of the first prospective randomized studies showed a positive effect of GH administration on delivery and live birth rates in women aged >40 years treated by intracytoplasmic sperm injection (ICSI) (2). Since then GH was used mainly in older women. However, more recent studies have suggested the possibility of a beneficial effect of GH on *in vitro* fertilization (IVF) outcomes even in some younger women with previous unexplained IVF failures (3). Moreover, the administration of GH to women receiving embryos from donated oocytes improved results in some cases, showing that GH can also have a positive effect on uterine receptivity (4).

GH is mainly secreted by the pituitary gland, but local production in various organs, including the ovary, has also been demonstrated (5). In addition to acting through its own receptors, some effects of GH can also be mediated by GH-induced insulin-like growth factor-I (IGF-I) gene activation (6).

The aim of this Research Topic was to analyze the effects of GH on the female reproductive function in different clinical scenarios, with a special accent on distinguishing those women who would benefit from GH administration from those who would not. Even though all biological and clinical aspects of GH action in the female reproductive system still remain unknown, the data presented in this series of papers have brought us closer to rational strategies of GH indication replacing purely empirical and blind indications.

## The Main Points of Individual Contributions

This series includes 13 papers: five original research articles and eight review articles, three of which focus on the complexity of GH physiology. In this section, they are referred to in a chronological order as they were published in the Journal. In their review article, Dosouto et al. present data from animal models and clinical trials showing that both GH and IGF-I are synthesized locally in the ovary, and the synthesis of IGF-I can be stimulated locally by other factors than GH, such as steroid hormones and gonadotropins. The original research paper by Li et al. shows that the addition of GH (200 ng/ml) to the culture medium improves oocyte maturation from the germinal vesicle stage to the metaphase II stage as well as their postfertilization developmental competence. The review article by Devesa and Caicedo highlights the potential role of ovarian angiogenesis as a mediator of GH effects on follicular development and oocyte quality. Xu et al. review scientific reports on the effect of GH on IVF outcomes, focusing on differences between those reporting a positive effect (mostly related to oocyte and embryo quality) and those in which no clinical benefit was demonstrated. The original research article by Shi et al. compares a retrospective single-center cohort analysis of 18,455 IVF cycles, including the transfer of fresh and frozen embryos, performed in good and low prognosis patients, and analyze how the cumulative live birth rate is influenced by the patient’s age, antral follicle count, and the number of oocytes obtained, respectively. This is one of three articles applying the Poseidon algorithm in defining poor-prognosis patients with poor ovarian reserve. Cai et al. present an original research article analyzing, retrospectively, the effect of GH in patients with poor ovarian reserve defined by the Poseidon algorithm. They show that GH pretreatment elevates ovarian response to stimulation, improves live birth rate, and reduces miscarriage rate in this group of patients. The review article by Yovich et al. compares their own experience at the PIVET Center (Australia) with that of the total of 42 GH studies performed since the year 2000 all over the world. They conclude that GH increases both oocyte and embryo utilization rates in most cases, but only ~50% are followed by elevated live birth rates. Lee et al., in their original research article, demonstrate that even low dose GH adjuvant treatment improves pregnancy outcomes in poor responders provided that it is combined with an ultra-long ovarian stimulation protocol, particularly in women under 40 years of age. In their review article, Yovich et al. resume the modes of action of GH and IGF-I in the ovary and suggest GH/IGF deficiency as the main cause of decreasing fertility in older women. They also add some original data suggesting how women needing GH support can be identified. The review article by Ipsa et al. presents a comprehensive overview of the molecular mechanisms of GH and IGF action in reproductive tissues, pointing out different interesting topics for future research. Two mini-reviews authored, respectively, by Altmäe and Aghajanova and Liu et al. deal with the effect of GH on endometrial receptivity. The former analyzes the potential molecular mechanisms of GH action in the endometrium, while the latter focuses on the current clinical experience. The last article of this series is an original research article by Tesarik et al. showing that GH administration also improves oocyte, zygote, and embryo quality, as well as the clinical IVF outcomes, in young women with previous repeated implantation failures. Although not included in this Research Topic, we would add the contemporary publication of Regan et al (7). which reported clear beneficial effects of GH on various receptors for reproductive hormones, as well as improving the profile of expression of these receptors in association with successful clinical outcomes in older women.

## Synthetic View and Conclusions

Taken together, the data presented in this Research Topic touch almost all aspects of GH effects on female fertility, both the biological ones and the clinical ones. Some of these data help identify women who are likely to benefit from GH treatment and distinguish them from those who are not. Other data show the ways for future biological and clinical research to further improve the scientific basis of GH indication.

## Author Contributions

All three authors (editors) contributed equally to the manuscript preparation. All authors contributed to the article and approved the submitted version.

## Conflict of Interest

The authors declare that the research was conducted in the absence of any commercial or financial relationships that could be construed as a potential conflict of interest.
